# Mucosal immune responses to SARS-CoV-2 infection and COVID-19 vaccination

**DOI:** 10.1016/j.vaccine.2025.127175

**Published:** 2025-05-22

**Authors:** Mathew J. Paul, Mohammed T. Hudda, Scott Pallett, Elisabetta Groppelli, Eugenia Boariu, Nicole Falci Finardi, Rachel Wake, Nidhi Sofat, Kathryn Biddle, Soraya Koushesh, Louis Dwyer-Hemmings, Richard Cook, Julian K-C. Ma

**Affiliations:** a Institute for Infection and Immunity, City St. George's University of London, Cranmer Terrace, London SW17 0RE, UK; b Department of Population Health, Dasman Institute, Jasim Mohamad Al Bahar St, Kuwait City, Kuwait; c Centre of Defence Pathology, Royal Centre of Defence Medicine, Queen Elizabeth Hospital Birmingham, Birmingham, B15 2WB, UK; d Faculty of Dentistry, Oral & Craniofacial Sciences, Kings College London, Floor 22, Guy's Tower, Guy's Hospital, Great Maze Pond, London, SE1 1UL, UK; e St. George's University Hospitals NHS Foundation Trust, Blackshaw Road Tooting, London SW17 0QT, UK

**Keywords:** Mucosal immunity, Saliva, Oral fluid IgA, COVID-19, Vaccination, Immune response, SARS-CoV-2, Neutralising antibody

## Abstract

SARS-CoV-2 continues to circulate in the community. We hypothesise that mucosal immunity is required to prevent continuing viral acquisition and transmission.

**Objectives:**

To determine whether SARS-CoV-2 infection or vaccination elicits specific neutralising antibodies in saliva, and to assess the longevity of protection.

**Methods:**

Initially, 111 COVID-19 convalescent participants were recruited, 11–369 days after diagnosis. Saliva and blood samples were assayed for antibodies specific for Spike protein, Receptor Binding Domain and Nucleoprotein. In a second cohort, 123 participants were recruited. Saliva and serum antibodies to the same antigens were assayed before and after their first and second COVID-19 vaccinations, with 150 day follow up.

**Results:**

Natural infection induces and boosts IgA and IgG in oral fluid and serum; vaccination does not induce or boost specific saliva IgA; IgG can be found in saliva after vaccination, but only when serum IgG concentrations are high; IgA is important for SARS-CoV-2 neutralisation activity by oral fluid, but there can also be contributions from serum IgG and other factors.

**Conclusions:**

New COVID-19 vaccines should target both systemic and mucosal immunity, to establish a first line of immune defence at the mucosal barrier. This would benefit vulnerable patient populations and may help to eradicate SARS-CoV-2 circulation.

## Highlights

• The relative importance of mucosal compared with systemic antibody responses in COVID-19 infection is not established.
• There is no clear consensus whether COVID-19 vaccination can induce or boost mucosal antibody responses.
• We demonstrate that natural infection induces and boosts IgA and IgG in saliva and serum, whereas vaccination only induces serum antibodies.
• IgA is important for SARS-CoV-2 neutralisation activity by saliva, but other factors, including IgG derived from gingival fluid and serum can contribute.
• New COVID-19 vaccines should target induction and boosting of mucosal immunity to maximise immune protection against SARS-CoV-2.

## Graphical abstract

**Figure:**
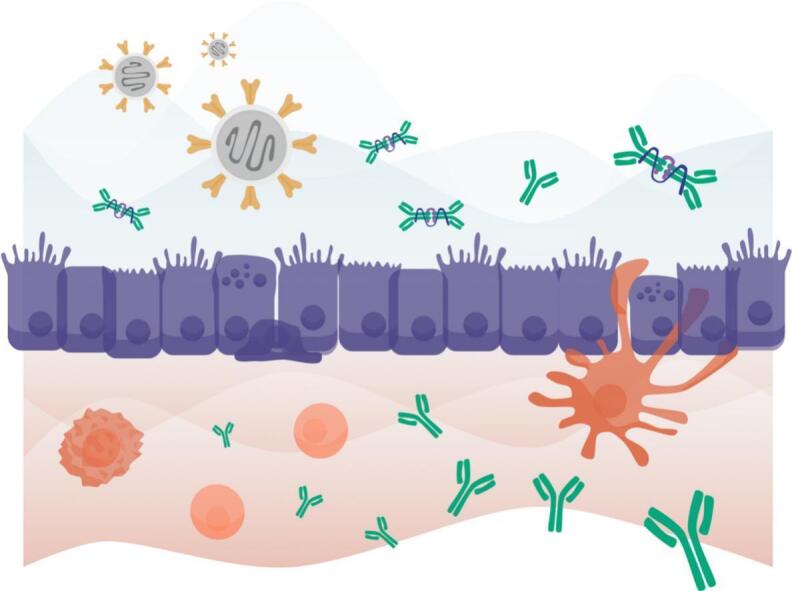
Optimal immunological protection against pathogens would be gained at mucosal surfaces by achieving external secreted mucosal antibodies as well as internal systemic antibodies.

## 1 Introduction

The COVID-19 pandemic was an unusual event, which provided an unprecedented opportunity to study de novo systemic and mucosal immune responses in a large adult population, and to compare the immune response to infection with that against immunisation.

Transmission of SARS-CoV-2 is mediated through mucosal tissues [1], so mucosal immunity and the induction of the specialised SIgA antibody in secretions [2] should be desirable. Many early studies reported specific mucosal IgA antibodies in infected and convalescent patients, including the presence of neutralising IgA in bronchoalveolar lavage and saliva samples [3]. IgA was also reported in nasal secretions within 1 week of symptom onset [4,5] and linked to better resolution of symptoms [6]. Mucosal antibodies have been reported to persist for at least 3 months post infection in saliva [7] and for up to 9 months in the nasal cavity [6,8,9].

The commercially approved SARS-CoV-2 vaccines all target the systemic immune response [10] and the question whether vaccination affects mucosal immunity remains unclear. Dogma would suggest that immune induction through mucosal associated lymphoid tissues [11] is required, however, some reports suggested that SARS-CoV-2 vaccination can both induce and boost mucosal antibodies. Ketas et al. reported specific IgA antibodies in patients' saliva after the first and second COVID-19 mRNA vaccinations respectively [12]. There have been similar reports from other groups [13]. More commonly, COVID-19 vaccination has been reported to boost mucosal antibodies in previously infected individuals [[14], [15], [16]]. However, other studies do not support the suggestion that COVID-19 vaccination stimulates mucosal antibodies in either naïve or convalescent individuals [[17], [18], [19]] even after the fourth booster vaccination [20].

In this study, we measured specific IgG and IgA responses in blood and saliva, and SARS-CoV-2 neutralising activity in saliva, in SARS-CoV-2 convalescent patients and for up to 5 months in naïve and convalescent patients receiving first and second COVID-19 vaccinations. SARS-CoV-2 continues to circulate in the community [21], and remains a concern for those with weakened immunity, for example through age or co-morbidities [22]. This study responds to increasing interest in boosting mucosal immunity, to create an additional protective barrier at the site of viral infection [23] [24].

## 2 Materials and methods

### 2.1 Public involvement

Members of the public (*n* = 4) were involved during the protocol development stage, considering the logistics and practicalities of recruitment and sample collection. Prior to ethics review, the proposal was reviewed by public volunteers (*n* = 3) and further recommendations were incorporated with respect to recruitment and conduct of the study.

### 2.2 Ethics statement

The MuCOVID trial (An investigation of quality and longevity of mucosal immune responses to SARS-CoV-2 infection; IRAS project ID: 287928) received ethical approval (REC reference: 20/EM/0227) and was approved by the UK NHS Health Research Authority, HRA and Health and Care Research Wales (HCRW). The trial was sponsored by St. George's University of London.

### 2.3 The MuCOVID trial

The aim was to compare mucosal and serum antibody responses against SARS-CoV-2 after infection and vaccination. Participants were recruited from staff, students and patients at St George's University Hospitals NHS Foundation Trust and St. George's University of London. The inclusion criteria were ≥ 18 years of age; and willing and able to give written informed consent. The exclusion criteria were uncontrolled infection; lacking capacity for comprehension of procedures required in participation and consent; and female participants who were or suspected to be pregnant.

### 2.4 Samples and sample processing

Peripheral venous blood was collected into SST II vacutainer tubes (BD). After clotting at room temperature for 30 min, serum was separated by centrifugation, aliquoted and stored at -20 °C.

Whole unstimulated saliva (oral fluid) was collected by asking the patient to salivate directly into a sterile container containing 5 μl Nonidet-P40, for up to 5 mins or until a volume of 5 ml was collected as described previously [25]. Samples were kept on ice for up to 6 h before processing. The saliva was clarified by centrifugation at 4863 RCF and 4 °C for 16 min (Hettich Rotina 380R), aliquoted and stored at -20 °C.

### 2.5 SARS-CoV-2 antigens

Details of SARS-CoV-2 recombinant Spike, receptor binding domain (RBD) and Nucleoprotein (NP) are given in Supplemental Materials.

### 2.6 ELISA procedures

ELISA assays to quantify serum IgG or IgA and saliva IgA or IgG antibodies to Spike, RBD or NP were developed and are described in Supplemental Material. Serum IgG ELISAs against Spike were optimised using a panel of 10 sera from SARS-CoV-2 sero-positive patients and 10 sera from SARS-CoV-2 sero-negative patients kindly provided by Dr. Tim Planche (South West London Pathology). All serum antibody assays were subsequently tested against a panel of 100 serum samples collected prior to October 2019, kindly provided by Dr. Henry Staines (St. George's Univ. of London).

### 2.7 Binding antibody units (BAU) calculation

Two convalescent antiserum samples (IHS328 and IHS230) were calibrated against the First WHO International Standard for anti-SARS-CoV-2 immunoglobulin (human), as described [26,27].

An arbitrary value of 1000 BAU/ml is assigned to the WHO standard when comparing antibodies of the same class and specificity. Standard curves on each assay plate were fitted using a 4-parameter logistic model (Graphpad Prism). Sample readings were averaged and sample BAU values were interpolated using a sample dilution corresponding to the linear part of the assay plate standard curve. BAU values were calculated to 3 d.p.

### 2.8 Saliva neutralisation assay

Details of Vero-AT cells and SARS-CoV-2 challenge virus are given in Supplemental Materials. For plaque reduction neutralisation test (PRNT), Vero-AT cells were seeded to obtain confluent monolayers (10^5^ cells/well; 12-well plate, Nunc) and allowed to settle overnight. Saliva samples were serially diluted (1:2) in media supplemented with 2 % FCS and Amphotericin B (0.50 μg/ml, Thermo Fisher Scientific) and incubated for 1 h at 37 °C with 40 pfu of SARS-CoV-2. After incubation, the virus-saliva mixture was transferred onto a confluent monolayer of Vero-AT cells and allowed to adsorb for 60 min at 37 °C. The inoculum mixture was removed and replaced with an overlay of 0.8 % Avicel (Sigma) in growth medium. The monolayers were incubated at 37 °C, 5 % CO_2_ for 48 h, then fixed and stained with paraformaldehyde 10 % (Sigma) and crystal violet (1×, Sigma) in PBS. Plaques were counted and neutralisation expressed as % of a non-neutralising control sample of saliva.

### 2.9 Statistical analysis

Statistical analyses were conducted in Stata version 18. Due to the skewed distributions of the antibody variables of interest in this study, all variables were assessed in terms of medians and inter quartile ranges. Details of the tests used are in Supplemental Materials. The 1 % and 5 % significance levels were used for all testing.

## 3 Results

### 3.1 The MuCOVID trial recruitment

234 participants (114 male; 120 female) were recruited.

Cohort A - 111 participants (53 male; 58 female) were diagnosed COVID-19 positive by RT-PCR. The median age was 56.0 years (range 23.6–88.3 years). A single sample of blood and saliva was collected; the range was 11–369 days after diagnosis.

Cohort B - 123 participants (61 male; 62 female) were recruited between January 2021 and November 2022. The median age was 46.8 years (range 22.2–88.4 years). Depending on the time of recruitment, samples were collected on up to 6 occasions; before vaccination (*n* = 29), at 3–5 weeks (*n* = 56) and 8–10 weeks (*n* = 58) after first vaccination, and at 3–5 weeks (*n* = 97), 8–10 weeks (*n* = 83) and 20 weeks (*n* = 82) after second vaccination. 97 participants received two doses of the Pfizer vaccine, 21 participants received two doses of the Oxford/AstraZeneca vaccine, 1 received two doses of Moderna vaccine, 1 received two doses of the Sinovac vaccine, 1 received a first dose of the Oxford/AstraZeneca vaccine followed by a second dose of the Pfizer vaccine, and two participants chose not to be vaccinated. The cohort was divided into a “No COVID history” group (*n* = 69) and a “COVID history” group (*n* = 54) according to a consistent history of symptoms or a positive test by either RT-PCR or lateral flow device.

If a participant contracted COVID-19 during the sampling period, samples taken after infection were excluded from the analysis.

### 3.2 COVID-19 infection induces serum IgG and mucosal IgA antibodies against SARS-COV-2 Spike protein for at least 6 months

**Fig. 1:**
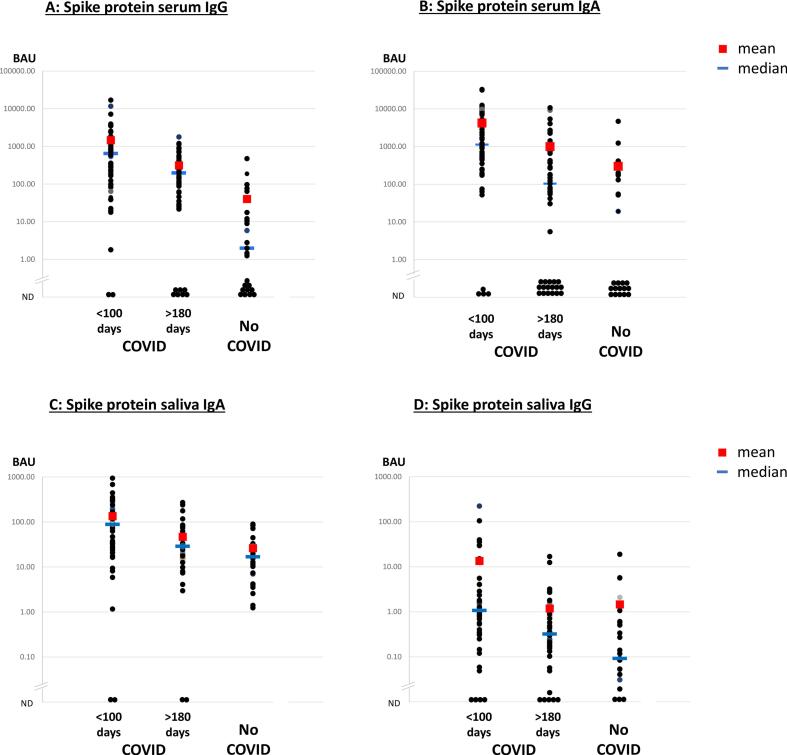
Serum and mucosal IgG and IgA antibodies against SARS-CoV-2 Spike protein following COVID-19 infection. A: Serum IgG; B: Serum IgA; C: Saliva IgA; and D: Saliva IgG. Specific antibodies were measured by Spike antigen capture ELISA and the binding antibody units (BAU) calculated according to the first WHO International Standard. 3 groups are shown, samples taken less than (<) 100 days or more than (>) 180 days after COVID-19 diagnosis, and samples from participants with no history of COVID-19 (No COVID). ND = not detected. Means are shown by red squares, medians are shown by blue lines. The serum results shown for each individual represent the mean from duplicate wells, whereas the saliva results shown for each individual represent the mean of at least 3 replicate assays. (For interpretation of the references to colour in this figure legend, the reader is referred to the web version of this article.)

Serum and saliva antibodies from Cohort A participants within (<) 100 days of diagnosis or greater than (>) 180 days were compared against patients with no history of COVID-19 (No COVID) from Cohort B, sampled before vaccination. The anti-Spike protein results are shown in Fig. 1.

Specific IgG antibodies were detected in 54/56 (96 %) serum samples in the COVID <100 days group compared with 15/25 (60 %) in the No COVID group (Fig. 1A). As expected, average anti-Spike serum IgG is significantly higher in samples taken <100 days after COVID-19 diagnosis compared with the No COVID group, the difference in the median antibody levels was over two orders of magnitude (*p* < 0.01). In samples taken >180 days after COVID-19, specific IgG antibodies were detected from 49/56 (86 %) participants and the average IgG levels were significantly higher than the No COVID group (p < 0.01). Average IgG antibodies levels in >180 day samples were significantly lower than in <100 day samples (p < 0.01). Similar patterns were observed for anti-Spike serum IgA antibodies (Fig. 1B). Serum IgA levels in real terms, were lower than serum IgG. This is not evident when measured as BAUs, however the median antibody end-point titre for anti-Spike serum IgG in the <100 days group was 25,600, whereas for anti-Spike serum IgA it was 1600 (data not shown).

Similar patterns were seen for anti-Spike saliva IgA (Fig. 1C). Specific saliva IgA was detectable in almost all samples, but average antibody levels <100 days after COVID-19 diagnosis were significantly higher than in the >180 days COVID group and the No COVID group (both *p* < 0.01). There was a borderline significant difference (*p* = 0.05) comparing the COVID >180 days group with the No COVID group. The specific IgA median end-point titre in saliva, was 128 in the <100 days COVID group and 32 in the >180 days COVID group (data not shown).

Anti-Spike IgG antibodies were also found in saliva (Fig. 1D). Again, the average saliva IgG level in the <100 days COVID group was significantly higher compared with both the >180 days COVID and the No COVID groups (p < 0.01). However, there was no significant difference between antibody levels in >180 days COVID group compared with the No COVID group. The median saliva IgG antibody end-point titres were 128 in the <100 days COVID group but just 8 in the >180 days COVID group (data not shown).

In order to estimate the longevity of antibody responses in saliva, the >180 days after COVID” group was divided into three sub-groups, 180–229 (*n* = 22), 230–279 (*n* = 14) and 280–330 (*n* = 10) days. Saliva IgA levels >20 BAU were found in 73 %, 71 % and 40 % of participants in these groups respectively. Saliva IgG levels >1 BAU were found in 25 %, 21 % and 10 % of participants in these groups respectively.

A similar pattern of serum and saliva antibody results was found against NP (Suppl. Fig. 1) and RBD (not shown).

### 3.3 COVID-19 vaccination does not induce specific mucosal IgA antibodies

**Fig. 2:**
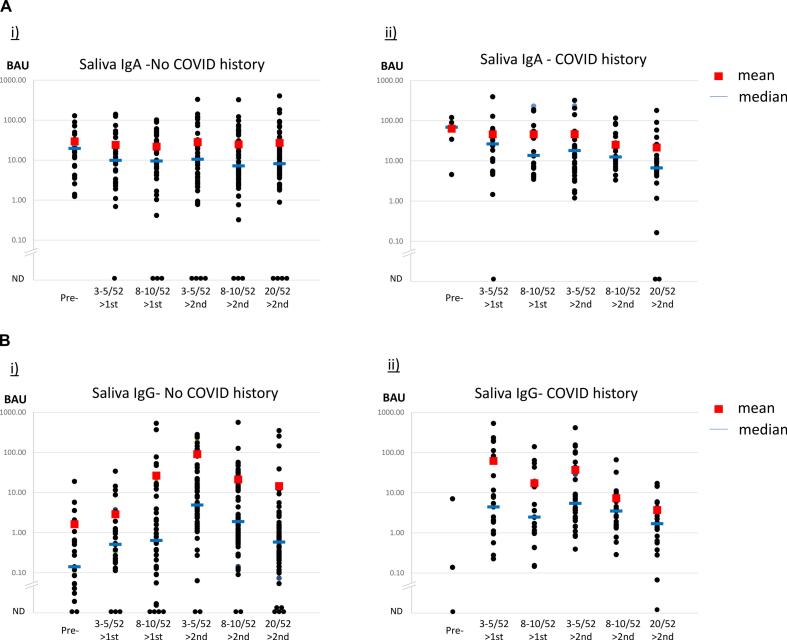
Spike protein saliva IgA and IgG before and after first and second immunisations. A: Saliva IgA; B: Saliva IgG. Specific antibodies were measured by Spike antigen capture ELISA and the binding antibody units (BAU) calculated according to the first WHO International Standard. Results of samples from participants with no history of COVID-19 (No COVID) and from participants with confirmed COVID history are shown separately. Antibodies were measured at 6 time points, once before immunisation, twice (at 3–5 weeks and 8–10 weeks) after the first immunisation and three times (at 3–5 weeks, 8–10 weeks and 20 weeks) after the second immunisation. ND = not detected. Means are shown by red squares, medians are shown by blue lines. The serum results shown for each individual represent the mean from duplicate wells, whereas the saliva results shown for each individual represent the mean of at least 3 replicate assays. (For interpretation of the references to colour in this figure legend, the reader is referred to the web version of this article.)

Fig. 2A shows saliva IgA to Spike protein before and after first and second immunisations. In participants with No COVID history, the median anti Spike saliva IgA level was 19.6 BAU before vaccination (*n* = 26), which decreased to 9.9 BAU 3–5 weeks after the first vaccination (*n* = 30) and remained around that level in subsequent samples, 8–10 weeks after first vaccination (*n* = 44) or in the three samples following second vaccination (*n* = 60). There was no difference in saliva IgA levels comparing samples taken 3–5 weeks after first and second vaccinations.

In participants with COVID-19 history, only 5 participants were recruited before the first immunisation, with a median anti Spike saliva IgA level of 68.74 BAU. There was no significant change in samples taken 3–5 weeks (*n* = 23) or 8–10 weeks after the first vaccination (*n* = 22), or 3–5 weeks following second vaccination (*n* = 36). The drop in saliva IgA 8–10 weeks (n = 23) and 20 weeks (*n* = 24) after the second vaccination did reach significance compared with earlier samples (*p* < 0.05).

Fig. 2B shows saliva IgG levels against Spike protein before and after first and second immunisations. The timing of samples and numbers at each time point were the same as for saliva IgA to Spike protein (above). In the No COVID groups, the IgG antibody levels were low, but the changes in median BAU levels over the sampling period are consistent with vaccination, i.e. a rise in average antibody levels following first vaccination and a greater rise following second vaccination, followed by a wane in antibody over the study period. The differences between adjacent samples are only significant however, between 8 and 10 weeks after first vaccination and 3–5 weeks after second vaccination, and subsequent samples when the antibody level wanes (*p* < 0.05). In participants with COVID history, only three samples were collected before vaccination. The average saliva IgG level after first vaccination was similar to levels in No COVID participants, 3–5 weeks after second vaccination, which is consistent with a booster, rather than a priming, response. An increase in antibody levels between samples at 8–10 weeks after first vaccination and 3–5 weeks after second vaccination was significant (p < 0.05), but peak antibody levels after first and second vaccination were equivalent. As before, a significant wane in antibody levels was observed after second vaccination.

At 8 weeks after second vaccination and beyond, the saliva IgG antibody levels were consistent with those observed in the <100 days COVID convalescent group (Fig. 1B).

Serum IgG and IgA seroconversion was confirmed in all Cohort B participants, with results demonstrating a typical primary and secondary antibody response in participants with No COVID history and an elevated immune response to first vaccination in those with prior COVID history (Supplemental Figs. 2 and 3, Supplemental Table 1).

The anti-Spike serum and saliva antibody results were consistent with those found against NP and RBD (not shown).

### 3.4 SARS-CoV-2 virus neutralisation by saliva is greater in COVID-19 convalescent participants than those vaccinated against COVID-19 with No COVID history

**Fig. 3:**
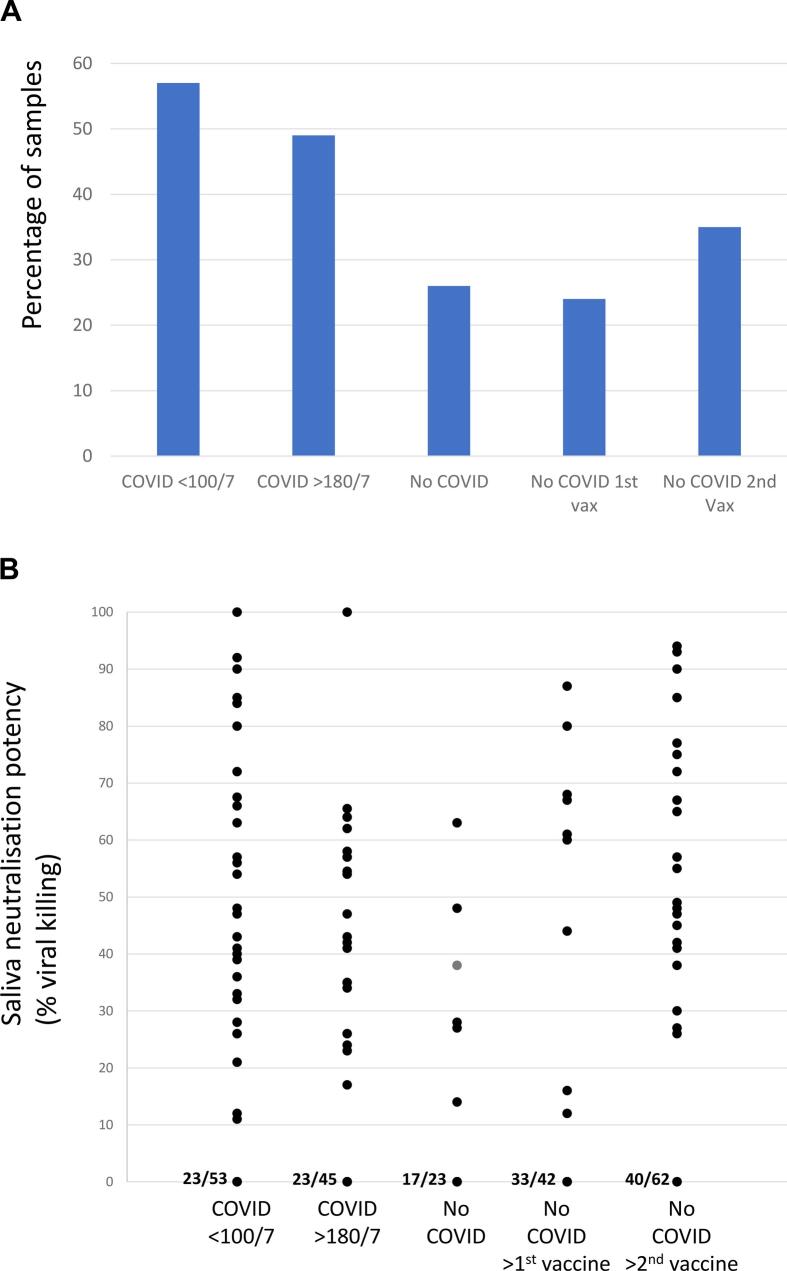
Virus neutralisation by saliva in COVID convalescent patients and patients with no history of COVID, before and after immunisation. A) Percentage of participants' samples with SARS-CoV-2 neutralising activity in each of 5 groups. COVID (convalescent participants) at <100 days or > 180 after diagnosis, and No COVID (participants with no history of COVID) samples taken before 1st vaccination, 3–10 weeks after first vaccination and 3–10 weeks after second vaccination; B) Virus neutralising potency in positive samples in the same 5 groups. The potency of saliva virus neutralising activity is represented by % viral killing in a FAVN assay. On the x-axis, the proportion of samples with no detectable neutralisation activity is shown (eg 23/53).

Saliva (oral fluid) samples from COVID-19 convalescent participants and No COVID participants, before and after vaccination were tested for SARS-CoV-2 virus neutralisation. The potency of the saliva samples' virus neutralising activity, represented by % viral neutralisation, are shown in Fig. 3.

Many samples had no detectable neutralising activity. But in Cohort A participants, 57 % of COVID <100 days samples and 49 % of COVID >180 days samples had SARS-CoV-2 neutralising activity, compared to 26 % samples in the No COVID pre-vaccination group (Fig. 3A). In Cohort B participants, the first vaccination resulted in no change; 21 % samples had neutralising activity, but this increased to 35 % samples after the second vaccination (Fig. 3A). The proportion of saliva samples with SARS-CoV-2 neutralising activity was significantly lower in the No COVID groups (before and after vaccination) compared with the COVID <100 days group (*p* < 0.05), but not compared with the COVID >180 days group.

The potency of SARS-CoV-2 virus neutralising activity in neutralising saliva samples, is shown in Fig. 3B. There was a trend towards greater potency of viral neutralisation in the COVID <100/7 group, but this did not reach statistical significance.

### 3.5 The SARS-CoV-2 immunological history of one participant

**Fig. 4:**
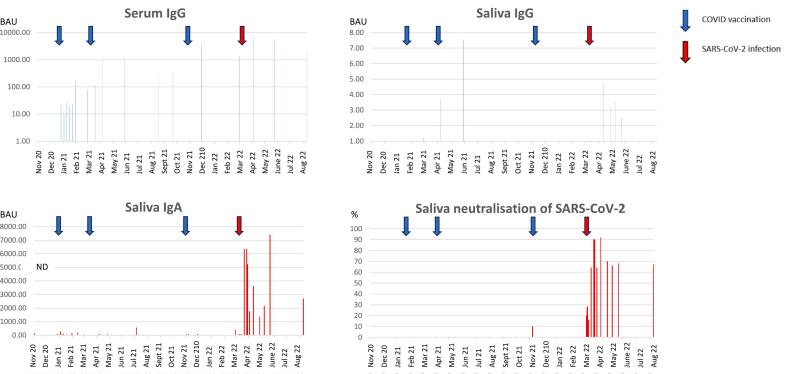
Serum IgG and Saliva antibody responses to COVID vaccination and SARS-CoV-2 infection in one individual between 10/11/2020–10/8/2022. 66 serum and saliva samples were collected over a 22 month period. Specific antibodies were measured by Spike antigen capture ELISA and the binding antibody units (BAU) calculated according to the first WHO International Standard. Spike protein specific serum IgG, saliva IgG and saliva IgA levels measured over 22 months are shown in individual graphs. Whole saliva neutralisation potency against SARS-CoV-2, measured by PRNT is shown in the fourth graph. The serum results represent the mean from duplicate wells, whereas the saliva results represent the mean of at least 3 replicate assays.

Multiple samples were taken from one participant between November 2020–August 2022. Sampling was intermittent and focused around SARS-CoV-2 challenges, but 66 samples were taken, covering all but 5 of the 22 months (Fig. 4). During that period, the individual received three Pfizer vaccinations in January, March and November 2021 (blue arrows), but eventually succumbed to COVID-19 infection, which was diagnosed by lateral flow assay in March 2022 (red arrow).

The first sample in November 2020 was seronegative for anti Spike IgG. Thereafter serum IgG was detected at every sample time point, and the response to vaccinations is evident. Serum IgG was further boosted by SARS-CoV-2 infection.

Saliva was collected at the same 19 time points. Additional saliva samples were collected as follows – Apr 2021 × 1, May 2021 × 2, Jul 2021 × 1, Nov 2021 × 4, Mar 2022 × 11, Apr 2022 × 3, and May 2022 × 3. Consistent with previous results, there was no significant increase in saliva IgA throughout the vaccination period, until SARS-CoV-2 infection in March 2022, and the levels then remained elevated until the last sample in Aug 2022.

Saliva IgG was detected intermittently after the second vaccination, and again after infection, but the levels were very low (single digit BAUs) and short-lived.

The SARS-CoV-2 neutralisation potency was measured for every saliva sample collected. No significant neutralisation was observed until after SARS-CoV-2 infection, and this was also sustained until the last sample in Aug 2022.

### 3.6 Evidence for muco-conversion in the absence of sero-conversion

**Fig. 5:**
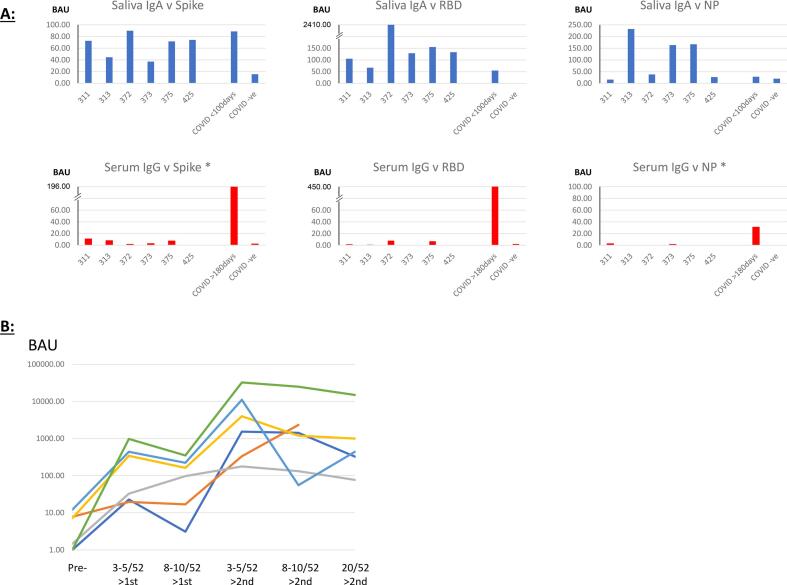
Saliva IgA antibodies to SARS-CoV-2 antigens in participants with no seroconversion or infection history. A: Saliva IgA and serum IgG levels to Spike, RBD and NP are shown for 6 participants with no history of COVID infection or immunisation. Specific antibodies were measured by antigen capture ELISA and the binding antibody units (BAU) calculated according to the first WHO International Standard. The positive and negative controls were for saliva antibodies were median values from 61 COVID convalescent participants (samples <100 days) and 21 COVID -ve participants, and for serum antibodies samples from 51 COVID convalescent participants (samples >180 days) and 25 COVID -ve participants. B: Spike protein specific serum IgG levels before and after COVID immunisation in 6 participants. Specific antibodies were measured by Spike antigen capture ELISA and the binding antibody units (BAU) calculated according to the first WHO International Standard. Antibodies were measured at 6 time points, once before immunisation, twice (at 3–5 weeks and 8–10 weeks) after the first immunisation and three times (at 3–5 weeks, 8–10 weeks and 20 weeks) after the second immunisation. The serum results shown for each individual represent the mean from duplicate wells, whereas the saliva results shown for each individual represent the mean of at least 3 replicate assays. Each line represents samples from an individual participant.

Six participants were found with saliva IgA antibodies to the three SARS-CoV-2 antigens at levels that were consistent with previous infection, but with no history of COVID-19 infection and no evidence for seroconversion, with very low or undetectable serum IgG antibodies to Spike (Fig. 5A) and NP (not shown). The saliva IgA levels to each antigen were equivalent to or greater than the median levels for COVID convalescent patients' samples taken up to 100 days after diagnosis (Fig. 5A).

To confirm that these 6 participants were systemically immunologically naïve, their subsequent serum IgG responses to vaccination were observed. In each case, the response was consistent with no previous exposure to SARS-CoV-2, as the anti-Spike IgG antibody levels after first vaccination were consistently boosted by over one log following second vaccination (Fig. 5B), similar to results shown in Suppl. Fig. 2 Panel i (No COVID), but not Panel ii (COVID).

## 4 Discussion

Our results confirm that mucosal immune responses are elicited in the majority (96 %) of infected patients, and can persist, with 71 % of convalescent patients remaining positive for Spike specific saliva IgA for between 230 and 279 days after infection. These results, in a large cohort of patients, support the findings of others, that SARS-CoV-2 infection induces mucosal and systemic antibody responses that are robust and detectable for at least 7 months [28].

We also demonstrate conclusively that saliva IgA antibodies are neither induced nor boosted by systemic COVID-19 vaccination. Vaccination can though, result in specific IgG antibodies in saliva, the levels of which reflect the levels of IgG in serum. Interestingly, the elevated serum IgA levels resulting from vaccination did not result in any change in saliva IgA. These results were supported in the longitudinal follow-up of a single individual, where SARS-CoV-2 specific saliva IgA was not detected over the course of three vaccinations, but only after infection.

Antibodies in mucosal fluids are predominantly secretory IgA antibodies that are mucosally derived. They may also include monomeric antibodies (IgG and IgA) derived from blood. In the mouth, these enter through gingival fluid [29]. Local gingival immune cells may also contribute [30], but we could not test for these in this study.

The functional significance of mucosal antibodies was demonstrated by SARS-CoV-2 neutralising activity in oral fluid. Both saliva IgA and serum IgG in oral fluid could be responsible for viral neutralisation and our neutralisation assay did not differentiate between the contributions of each antibody class. Variable levels of gingival disease would impact the leakage of serum antibodies into saliva and might be a factor in complicating the interpretation of saliva neutralisation activity against SARS-CoV-2 in the different patient groups.

Comparing the levels of different antibody classes in different body fluids is difficult. We assessed antibody end-point titres, which gives an indication of antibody concentration. These results suggest that the concentration of specific IgG in saliva is similar to IgA shortly after infection, but wanes at a quicker rate – 16-fold in the >180 days group, compared with 4-fold for IgA.

Another assessment of antibody concentration is based on function, ie virus neutralisation. We were not however, able to demonstrate a correlation between concentration of IgA, IgG, or IgA + IgG in our samples and viral neutralisation (data not shown), which probably reflects the technical difficulties associated with the assays involved. Similarly, in one study, low titres of nasal IgA antibodies in children were found to have comparable neutralisation capacity to higher titres in adults [31]. Variability in the collection of mucosal samples, the assessment of antibody levels and the neutralisation potency all contribute to difficulty in finding correlations. In addition, some neutralising activity might be associated with non-specific factors in saliva, for example components of the innate immune response [32], which we have not been able to separate out.

However, our results suggest that saliva IgA makes the most important contribution to neutralisation activity. Firstly, an increase in neutralising activity was only seen in No COVID vaccinees after two vaccinations, suggesting that low IgG levels in saliva after primary immunisation are inadequate. Secondly, in the convalescent cohort 1, there was only a modest reduction in the proportion of samples with neutralising activity between the <100 and > 180 days groups, that was more consistent with the 4-fold reduction in saliva IgA titre, than the 16-fold reduction in saliva IgG titre in the same samples. Finally, although only a single case was analysed, in a longitudinal study, SARS-CoV-2 neutralisation activity in saliva was not detected over the course of three COVID-19 vaccinations but was detected following COVID-19 infection and the appearance of saliva IgA antibodies.

We also report six cases where mucosal immunity was observed in the absence of seroconversion. This raises the possibility that mucosal immune protection against SARS-CoV-2 might be sufficient to prevent systemic infection. Similar cases have been reported before. 4 patients (20 %) of Spike protein–seronegative individuals, were identified as Spike protein mucosal IgA positive in one study [33]. The authors postulated that low-level antigen exposure might elicit a mucosal IgA responses only. Ketas et al., who identified a single equivalent patient, suggested that the mucosal antibodies represented a cross-reactive response and that they might initially have been induced by other seasonal coronaviruses [12]. Our findings here, that subsequent immune responses to vaccination were characteristic for systemically naïve patients favours the former hypothesis.

Although our results provide some clear conclusions, there are also some anomalous results. Over half of our No COVID patients had detectable anti-Spike IgG antibodies; there is variability in antibody levels within individuals in the same groups; and SARS-CoV-2 neutralising activity was observed in the No COVID control group. Observational studies during the COVID-19 pandemic were challenging and included biological factors which were not understood at the time. For example, asymptomatic COVID-19 infection was not generally diagnosed, complicating immune response studies. The prevalence of asymptomatic COVID has been reported at 15.6 %, rising to 27.7 % in children in one review of over 50,000 patients with confirmed COVID [34]. In another meta-analysis involving almost 30 M patients, the percentage of asymptomatic SARS-CoV-2 infections among populations tested for and with confirmed COVID, was 0.25 % and 40.50 % respectively [35]. A second major issue was the rapid wane in antibody levels observed after both SARS-CoV-2 infection and vaccination [36]. In particular, the more rapid drop in antibodies to SARS-CoV-2 nucleoprotein (NP) made it virtually impossible to identify patients who had previously had asymptomatic COVID, once the vaccination programme was established. For these reasons, identifying a negative COVID-naïve group presented difficulties.

The assessment of antibody levels in oral fluids is difficult and results are more variable than those from serum. Oral fluid collection is complicated by viscosity and dilution with variable amounts of water, according to the level of stimulation, for example from oral movements or the sight or thought of food. Here, we collected unstimulated oral fluid, which needs to be taken into account when comparing our results with other studies that collect stimulated oral fluids using a variety of techniques. We were also careful to introduce a minimum number of processing steps between sampling and assay to reduce the risk of antibody degradation [37].

Attention to mucosal immunity has become of increasing interest. We postulate that the failure of vaccination programmes to prevent virus circulation in the community is related to lack of mucosal immunity. The COVID-19 burden remains significant [21,38]. There are more hospitalisations for COVID-19 than other seasonal respiratory infections and WHO reported almost 10,000 deaths from COVID-19 at the end of 2023. Immunocompromised patients with reduced responses to COVID-19 vaccines bear a disproportionate burden of the residual effects of COVID-19 [39,40], and better solutions are urgently needed to address the concerns of these vulnerable populations.

The prospect of a primary mucosal barrier, backed up by secondary systemic immunity against mucosal infections has always been attractive [2,41]. Specific mucosal antibodies to fortify the mucosal barrier could be provided through different approaches, including mucosal vaccines [42,43] or passive immunisation with IgA monoclonal antibodies [25,44]. Protecting against viral acquisition at the external mucosal barrier may have additional benefits. One study suggested that specific mucosal IgA resulting from early SARS-CoV-2 infection, retained activity against omicron variants such as XBB.1, which are highly evasive of IgG neutralisation [31]. In addition, protecting mucosal surfaces against colonisation might result in reduced virus circulation within communities and an ultimate reduction in the development of new variants [31].

## CRediT authorship contribution statement

**Mathew J. Paul:** Writing – review & editing, Methodology, Investigation, Formal analysis, Data curation. **Mohammed T. Hudda:** Writing – original draft, Methodology, Formal analysis. **Scott Pallett:** Writing – review & editing, Resources, Project administration, Investigation. **Elisabetta Groppelli:** Writing – review & editing, Writing – original draft, Investigation, Funding acquisition, Formal analysis, Data curation. **Eugenia Boariu:** Writing – review & editing, Investigation. **Nicole Falci Finardi:** Writing – review & editing, Investigation. **Rachel Wake:** Writing – review & editing, Investigation. **Nidhi Sofat:** Writing – review & editing, Resources. **Kathryn Biddle:** Writing – review & editing, Investigation. **Soraya Koushesh:** Writing – review & editing, Investigation. **Louis Dwyer-Hemmings:** Writing – review & editing, Investigation. **Richard Cook:** Writing – review & editing, Investigation. **Julian K-C. Ma:** Writing – review & editing, Writing – original draft, Supervision, Resources, Project administration, Methodology, Investigation, Funding acquisition, Formal analysis, Data curation, Conceptualization.

## Declaration of competing interest

The authors declare that they have no known competing financial interests or personal relationships that could have appeared to influence the work reported in this paper.

## Data Availability

The data that has been used is confidential.

## Appendix A Supplementary data

Supplementary material 1 Image 1

Supplementary material 2 Image 2
