# Supplementary material for: Mucosal immune responses to SARS-CoV-2 infection and COVID-19 vaccination
Source: Vaccine. 2025 May 22;56:None. doi: 10.1016/j.vaccine.2025.127175 (PMC12286911; doi:10.1016/j.vaccine.2025.127175)
Supplement: Supplementary file 1 — Supplementary material 1 [file mmc1.docx]

Supplemental material

**SARS-CoV-2 antigens.**

Recombinant trimeric spike protein (Wuhan) produced in CHO was kindly provided by Dr. Svend Kjaer (Crick Institute). Recombinant receptor binding domain (RBD) of the SARS-CoV-2 (Wuhan) Spike (PDB: 6VYB, R319-F541) with a C-terminal 6xHis-tag was expressed in Expi293F cells using an expression vector kindly provided by Mark Dürkop (BOKU University, Vienna). Recombinant nucleoprotein (NP) was produced in *Nicotiana benthamiana* (Leaf Expression Systems, Norwich).

**ELISA assays:**

SARS-CoV-2 antigens were diluted to 2μg/ml (Spike and RBD) and 0.33μg/ml (NP) in phosphate buffered saline (PBS), pH7.4 with 0.1% sodium azide. ELISA plates (Nunc, MaxiSorp) were coated for 2 hours at 37^o^C. Blocking was with 5% BSA in dH_2_O /0.05% Tween 20, for 4 hours at 37^o^C. The plates were dried at room temperature (RT) and stored at 4^o^C for up to two weeks.

Serum aliquots were thawed at RT and diluted 1:100 in PBS (pH7.4). Saliva aliquots were thawed on ice, centrifuged at 13000rpm for 8 minutes at 4^o^C and diluted 1:2 in PBS (pH7.4). Samples were added to the ELISA wells in duplicate and a 4-fold dilution series. Positive control serum from a convalescent participant was included on all plates. A negative control serum or saliva from a COVID-19 naïve participant was included on serum and saliva assay plates respectively.

Incubation of samples was for 2 hours at 37^o^C for serum, or overnight at 4^o^C for saliva. The plates were washed with dH_2_O /0.05% Tween 20. Detection of bound antibodies was with either HRP Goat anti-human IgG Fc antiserum (AP113P, ThermoFisher) at 1:2000 dilution; or HRP Goat anti-human IgA alpha chain (ab97215, Abcam) at 1:4000 dilution in PBS (pH7.4) for 2 hours at 37^o^C. After washing, 3,3’ ,5,5’-tetramethylbenzidine (Thermo Fisher Scientific) substrate was added. The reaction was stopped with 2M sulphuric acid and plates were read at 450nm using an Infinite F200 Pro plate reader (Tecan).

**Saliva neutralisation assay reagents:**

Vero E6 cells expressing human ACE-2 and human TMPRSS-2 (Vero-AT) were obtained from NIBSC, UK, and grown at 37°C and 5% CO_2_ in Dulbecco-MEM supplemented with 10% heat inactivated foetal calf serum (Gibco, Thermo Fisher Scientific), penicillin (100 U/ml, Sigma), streptomycin (100 µg/ml, Sigma), Hygromycin B (250 µg/ml, Thermo Fisher Scientific) and G418 (250 µg/ml, Thermo Fisher Scientific). SARS-CoV-2 (England/2/2020) was obtained from Public Health England and passaged in Vero-AT cells. Virus stocks were quantified by plaque assay and titres expressed as plaque forming units per ml (pfu/ml).

**Statistical analysis**

The non-parametric test of the equality of medians test was utilised, where the null hypothesis is that the samples were drawn from populations with the same median, to assess differences in antibody levels in independent cohorts at different times relating to their exposure to SARS-CoV-2, and to assess whether anti-viral neutralisation potency in saliva was greater in convalescent patients compared with vaccinees.

To compare antibody responses over the course of the vaccination programme, we compared average values within individuals over time utilizing the sign test [27, 28]. We tested for differences in antibody levels for matched pairs of observations (i.e. observations within the same individuals across different time points in the immunisation schedule) where the null hypothesis is that the median of the differences between matched pairs is zero.

**Legend to Suppl. Figure 1:**

Serum and saliva antibodies from Cohort A participants within (<) 100 days of diagnosis or greater than (>) 180 days and from patients with no history of COVID-19 (No COVID) from Cohort B, sampled before vaccination. The anti-nucleoprotein (NP) results are shown.

**Legend to Suppl. Figure 2:**

Confirmation of systemic IgG immune response to SARS-CoV-2 vaccination.

Panel i) shows serum IgG to Spike protein before and after first and second immunisations in the No COVID history group. Priming and booster responses were observed after first and second vaccination respectively. The changes in antibody levels between each adjacent sample point were significant (p<0.05) and shown in Supplementary Table 1. The difference between antibody levels reached 3-5 weeks after the first and second vaccination was also significantly different (p<0.05).

Panel ii) shows serum IgG to Spike protein before and after first and second immunisations in participants with a COVID history. The anti-Spike antibody responses were consistent with a previous immunological encounter with SARS-CoV-2. Specific IgG levels after first vaccination were comparable to the levels in the No COVID group after second vaccination (panel i) and in these patients with previous exposure to SARS-CoV-2, the difference between antibody levels reached 3-5 weeks after the first and second vaccinations was not significant.

**Legend to Suppl. Figure 3:**

Confirmation of systemic IgA immune response to SARS-CoV-2 vaccination.

Panel i) shows serum IgA to Spike protein before and after first and second immunisations in the No COVID history group.

Panel ii) shows serum IgA to Spike protein before and after first and second immunisations in participants with a COVID history.
