# Supplementary material for: Mucosal immune responses to SARS-CoV-2 infection and COVID-19 vaccination
Source: Vaccine. 2025 May 22;56:None. doi: 10.1016/j.vaccine.2025.127175 (PMC12286911; doi:10.1016/j.vaccine.2025.127175)

# Suppl. Figure 1: Nucleoprotein serum IgG and saliva IgA

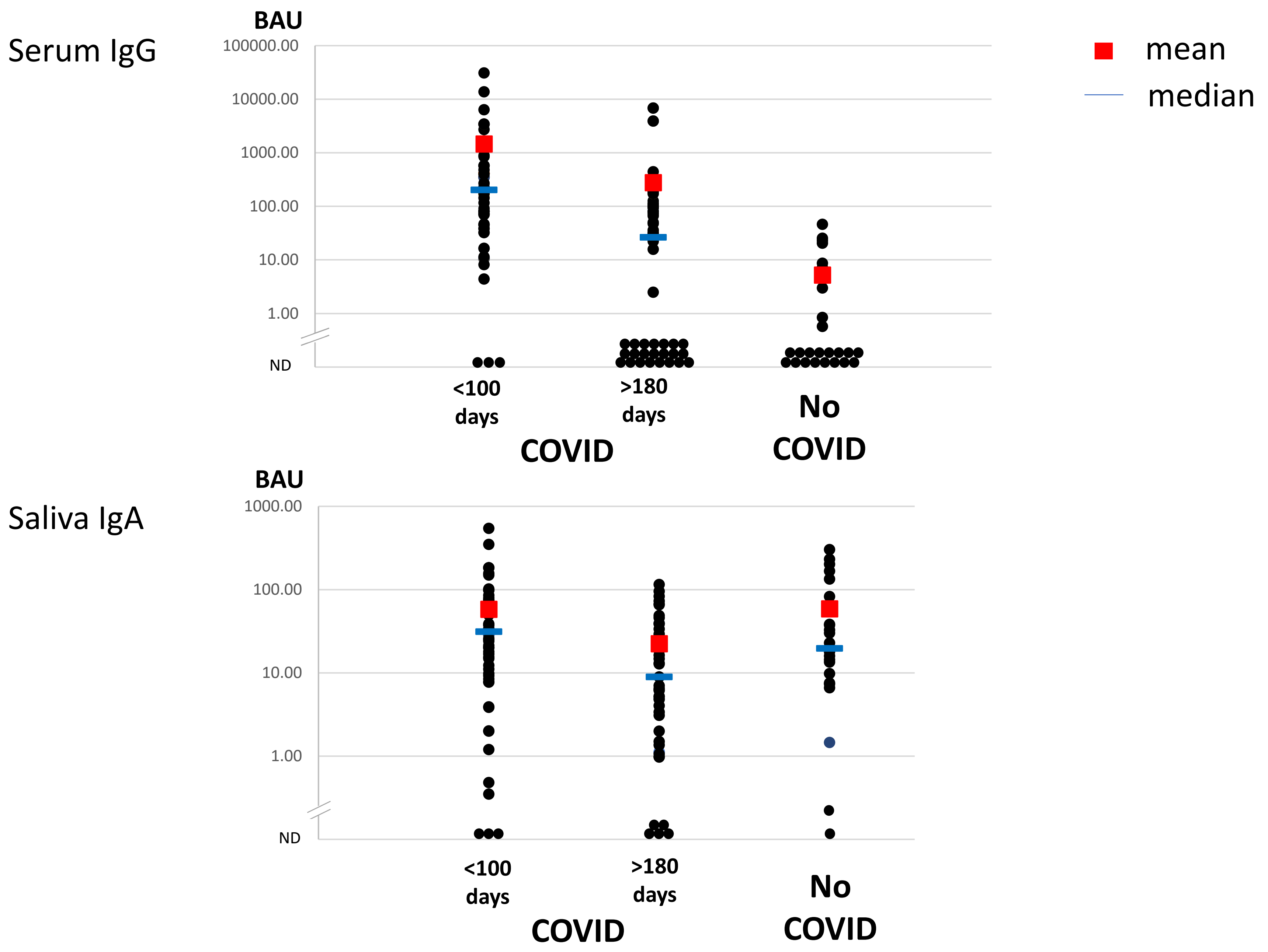

# Suppl. Figure 2: Spike protein serum IgG before and after first and second immunisation

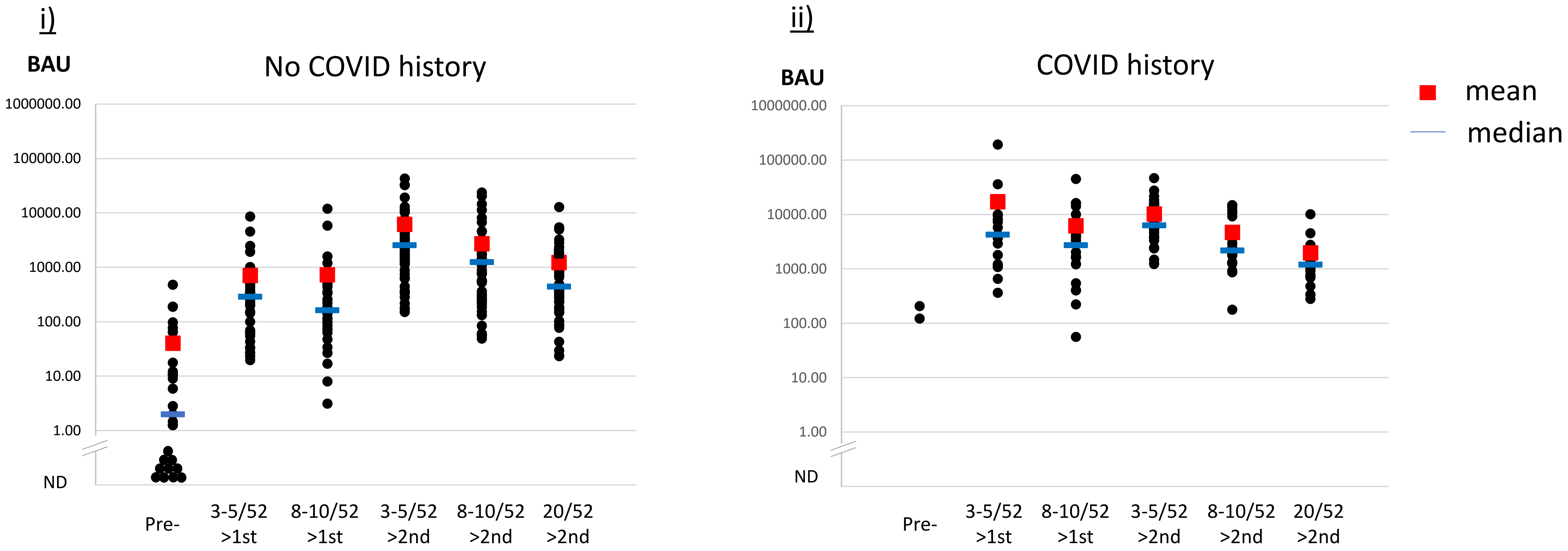

# Suppl. Table 1:

| No COVID | | n | Median | lower quartile - upper quartile | | | Difference in medians (p) |
|---|---|---|---|---|---|---|---|
| Spike serum IgG | T0 (**a**) | 25 | 2.00 | 0.10 - 41.90 | | | |
| | T1 (**b**) | 40 | 258.51 | 61.46 - 440.67 | | **a**/**b** | 0.000 |
| | T2 (**c**) | 34 | 150.92 | 72.58 - 349.60 | | **b**/**c** | 0.265 |
| | T3 (**d**) | 61 | 2473.03 | 1197.02 - 5054.24 | | **c**/**d** | 0.000 |
| | T4 (**e**) | 60 | 1171.74 | 328.2 - 2445.34 | | **d**/**e** | 0.000 |
| | T5 (**f**) | 59 | 399.38 | 207.8 - 1207.45 | | **e**/**f** | 0.000 |
| | | | | | | **b**/**d** | 0.000 |

| COVID | | n | Median | lower quartile - upper quartile | | | Difference in medians (p) |
|---|---|---|---|---|---|---|---|
| Spike serum IgG | T0 (**a**) | 2 | omit | omit | | | |
| | T1 (**b**) | 19 | 4186.62 | 1207.1 - 9585.57 | | **a**/**b** | |
| | T2 (**c**) | 21 | 2807.47 | 1211.51 - 4952.68 | | **b**/**c** | 0.109 |
| | T3 (**d**) | 34 | 7094.07 | 3802.29 - 14858.93 | | **c**/**d** | 0.022 |
| | T4 (**e**) | 21 | 2105.83 | 1275.15 - 3532.52 | | **d**/**e** | 0.001 |
| | T5 (**f**) | 21 | 1077.50 | 711.40 - 1510.21 | | **e**/**f** | 0.001 |
| | | | | | | **b**/**d** | 0.109 |

## Suppl. Figure 3: Spike protein serum IgA before and after first and second immunisation

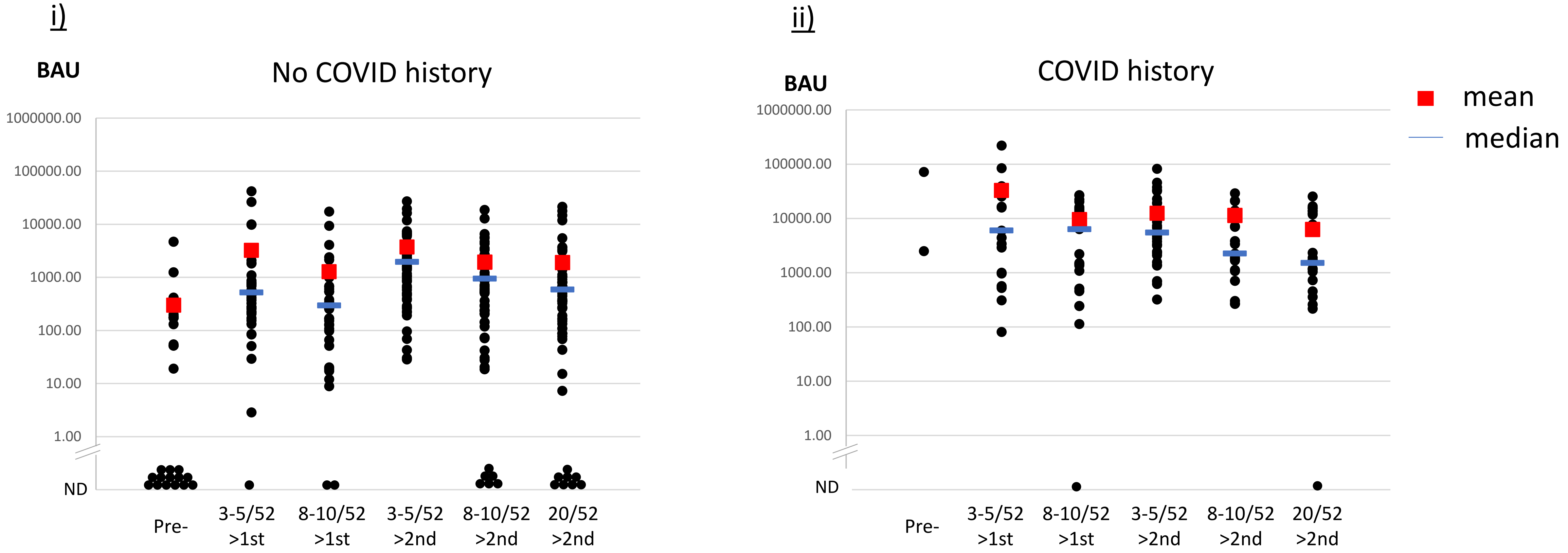

Supplement: Supplementary file 2 — Supplementary material 2 [file mmc2.pdf]
